# Vitrimer Nanocomposites for Highly Thermal Conducting Materials with Sustainability

**DOI:** 10.3390/polym16030365

**Published:** 2024-01-29

**Authors:** Younggi Hong, Munju Goh

**Affiliations:** Department of Chemical Engineering, Konkuk University, 120 Neungdong-ro, Gwangjin-gu, Seoul 05029, Republic of Korea; hongyg21@konkuk.ac.kr

**Keywords:** vitrimer, thermal conductivity, nanocomposite, sustainability

## Abstract

Vitrimers, as dynamic covalent network polymers, represent a groundbreaking advancement in materials science. They excel in their applications, such as advanced thermal-conductivity composite materials, providing a sustainable alternative to traditional polymers. The incorporation of vitrimers into composite fillers enhances alignment and heat passway broadly, resulting in superior thermal conductivity compared to conventional thermosetting polymers. Their dynamic exchange reactions enable straightforward reprocessing, fostering the easy reuse of damaged composite materials and opening possibilities for recycling both matrix and filler components. We review an overview of the present advancements in utilizing vitrimers for highly thermally conductive composite materials.

## 1. Introduction

Vitrimers are covalent adaptable network (CAN) polymers capable of undergoing dynamic covalent bond exchange reactions while maintaining their high crosslinking density [1,2,3,4]. They have received increasing attention as materials that combine thermosetting resins’ stability with thermoplastic resins’ processability. This innovative polymer is covalently bonded and possesses the unique ability to undergo dynamic bond exchange in response to external stimuli [5,6,7]. During this process, while CAN bonds break and form, a consistent bond density is maintained, allowing for a rearrangement of the high-topological network [8,9]. This bond exchange process proceeds slowly at room temperature, rendering vitrimers with mechanical properties similar to thermosetting resins. However, when the temperature surpasses the topological freezing transition temperature (T_v_), the bond exchange reaction accelerates, endowing vitrimers with thermoplastic-like characteristics, including reprocessing, remolding, and recycling [10,11,12].

The preparation of vitrimers requires the presence of dynamic covalent bonds capable of forming a CAN that facilitates exchange reactions. Hence, vitrimer systems exhibit the capacity for reversible reactions, allowing for the breaking and formation of bonds in response to external stimuli while maintaining the overall count of chemical bonds. This distinctive characteristic arises from the investigation and study of various dynamic covalent bonds, encompassing ester [13,14], carbonate [15], carbamate [16,17,18], acetal [19,20], imine [21,22,23], boron ester [24,25,26], diboroxine [27], silyl ether [28,29], disulfide [30,31], triazolium [32], and others [33], within this system.

**Figure 1:**
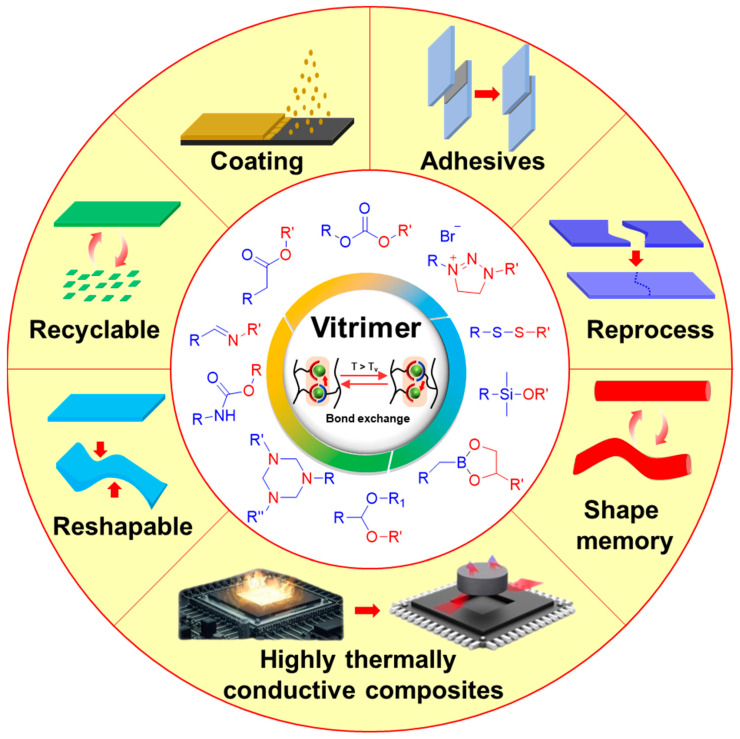
Diagrammatic representation of dynamic covalent linkages employed in vitrimer materials and their applications.

The versatility of vitrimers is underscored by their unique combination of stability and processability, leading to applications in reprocessable [34,35] and recyclable polymers [36,37] that contribute to sustainable practices. Additionally, vitrimers find use in coatings [38], adhesives [39,40], and reshapable polymers [41,42], showcasing easy processability for adjustments and repairs. Their shape memory properties offer utility in 3D printing [43,44,45]. Vitrimers play an especially significant role in the field of composites [46,47,48,49,50], showing a wide range of impacts in a variety of applications, thanks to their recyclability that traditional composite materials do not have. Consequently, vitrimers can serve as alternative materials to sustainable polymers currently used in composite materials. The versatile utilization of vitrimers across diverse fields highlights their broad impact on advancing scientific applications (Figure 1). Ongoing research endeavors are focused on exploring novel applications and optimizing the performance of vitrimer-based materials, contributing to sustained progress in materials science.

**Figure 2:**
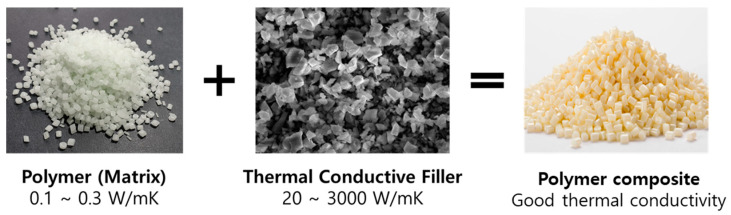
High thermal conductive polymeric composite materials are produced by integrating polymer and ceramic fillers with excellent thermal conductivity properties.

Recently, to propel the advancement of the next generation of compact, integrated, functional, and portable smart devices, swift and efficient heat dissipation is imperative. This is critical due to the substantial heat generated within these devices, which has the potential to adversely affect the safety and performance of electronic components throughout the operational lifespan of the device [51,52]. Polymer-based composites find extensive application as a solution to this issue. Polymer-based thermally conductive composite materials are fabricated with polymer (matrix) and high thermal conductive ceramic material (filler), as shown in Figure 2. Utilizing polymer composites with high crosslinking density and oriented fillers is an effective strategy for producing composites with superior thermal conductivity [53,54,55]. By enhancing these two factors, the mean free pathway of phonons is extended, minimizing phonon scattering and ultimately improving the thermal conductivity of the composite [56].

In this review, we present a study of recent advancements in a novel academic domain. Specifically, we focus on excellent thermal-conductivity composites while simultaneously utilizing the characteristics of vitrimers, such as reshaping and recyclability, thereby pursuing eco-friendliness. We explore the latest advancements in the field, emphasizing the potential for environmentally friendly solutions through the creation of composites with enhanced thermal properties and the distinctive characteristics of vitrimers.

## 2. Vitrimer-Assisted Filler Orientation for the Highly Thermal Conducting Pathway of Nanocomposites

**Figure 3:**
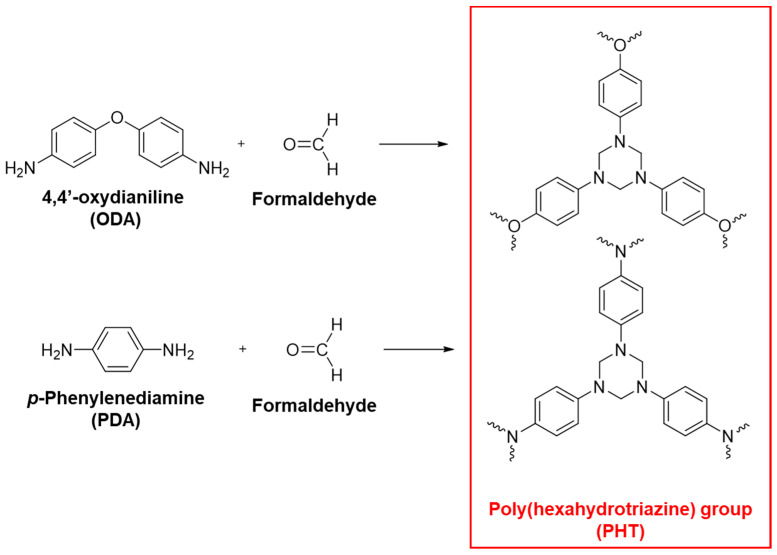
Synthetic routes for two types of poly(hexahydrotriazine) (PHT) polymers.

Research has been conducted on vitrimers used as high thermal conductivity nanocomposites, specifically focusing on composites involving the chemical bonding of 1,3,5-triazine. Within the 1,3,5-triazine chemical group, attention has been directed towards substances from the poly(hexahydrotriazine) (PHT) series. PHT, initially reported by IBM in 2014, is synthesized through the polycondensation of 4,4′-oxydianiline or *p*-phenylenediamine with paraformaldehyde (as shown in Figure 3), showcasing exceptional mechanical properties and mechanical strength, resulting from a high crosslink density [57,58].

**Figure 4:**
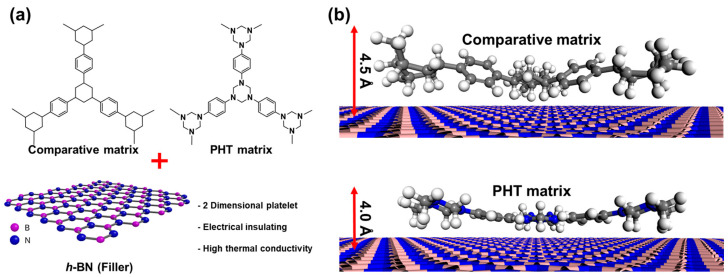
Geometry optimization is performed to model the interaction between nitrogen atoms in the PHT matrix and the *h*-BN surface. The comparative matrix involved examining the geometry-optimized structures of the PHT matrix and analogous molecules, replacing nitrogen atoms with carbon atoms near the *h*-BN surface, with oligomer units, for simplification, depicted in (**a**), and detailed views are provided (**b**). Reproduced from Ref. [61]. Copyright 2023, Elsevier.

In this review, a nanocomposite is fabricated by utilizing PHT synthesized by *p*-phenylenediamine with paraformaldehyde with vitrimer properties, and hexagonal boron-nitride (*h*-BN) is chosen as the filler due to its advantageous plate-like structure, proving superior to spherical fillers [59]. The interfacial affinity between the filler and the matrix is crucial for maximizing properties such as thermal conductivity while minimizing molecular voids [58,59,60,61]. A computational analysis is employed to assess the intermolecular affinity between PHT and the comparative matrix (the geometry-optimized structures of the PHT matrix and analogous molecules, replacing nitrogen atoms with carbon atoms) with *h*-BN, comparing the oligomeric units of each molecule. The analysis revealed a favorable interaction between the nitrogen in the matrix and the boron in *h*-BN (Figure 4), leading to the flattening of the overall molecular structure of PHT and a reduced molecular distance between the heteromolecules.

**Figure 5:**
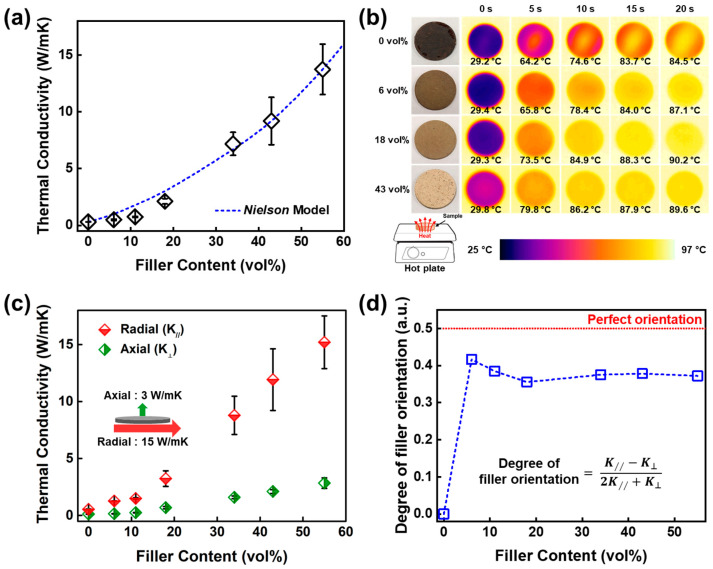
(**a**) The graph into the thermal conductivity of *h*-BN/PHT composites with varying loadings of *h*-BN fillers. A predictive model, based on the *Nielsen* model, is depicted as a dashed blue line in the same plot. (**b**) Thermal infrared images capture the heating process of the composites at 90 °C, with the filler volume fractions indicated in parentheses, and the corresponding temperature values are provided for each image. (**c**) The measured radial (K_//_) and axial (K_⊥_) thermal conductivities, along with their standard deviations, are presented to illustrate the anisotropy in thermal conductivity concerning *h*-BN loading. (**d**) Degree of filler orientation by calculated anisotropic thermal conductivity. The red dotted line serves as a reference for perfect *h*-BN orientation in the radial direction of the composite. Reproduced from Ref. [61]. Copyright 2023, Elsevier.

The isotropic thermal conductivity of the *h*-BN/PHT composite materials is measured, showing a gradual increase with the rise in *h*-BN content, as shown in Figure 5a. At the highest *h*-BN content, the thermal conductivity reaches 13.8 Wm^−1^K^−1^, aligning with the graphical representation of the *Nielson* model. The *Nielsen* model for thermal or electrical conductivity of composites is a predictive model that considers the influence of both the composition and structure of the composite material. It quantitatively models the conduction properties by incorporating factors such as the type and arrangement of components, providing a detailed understanding of how these parameters affect the overall conductivity of the composite. Consequently, this model indicates that enhancing the filler loading can improve thermal conductivity [59,61]. The *Nielsen* model is expressed as Equation (1):

(1) $$\frac{K_{c}}{K_{p}}=\frac{1+AD\varphi }{1-D\lambda \varphi }$$

where *K_c_*, *K_p_*, and *φ* are the thermal conductivities of the composite and the polymer matrix and the filler volume fraction, respectively. The geometry factors, A, D, and λ, relate to the filler orientation ratio, the maximum filler volume fraction, and a significant amount of voids. Following the *Nielsen* model suggests that the *h*-BN/PHT composite material easily achieves thermal conductivity within the suitable range of 2 to 8 Wm^−1^K^−1^ for high thermal dissipation applications. To visually demonstrate the enhanced thermal conductivity, a thermal IR image camera monitors the temperatures with different *h*-BN loadings (Figure 5b). Samples with higher thermal conductivity exhibit a faster temperature increase, indicating more efficient thermal energy conduction through these thermally conductive samples.

**Figure 6:**
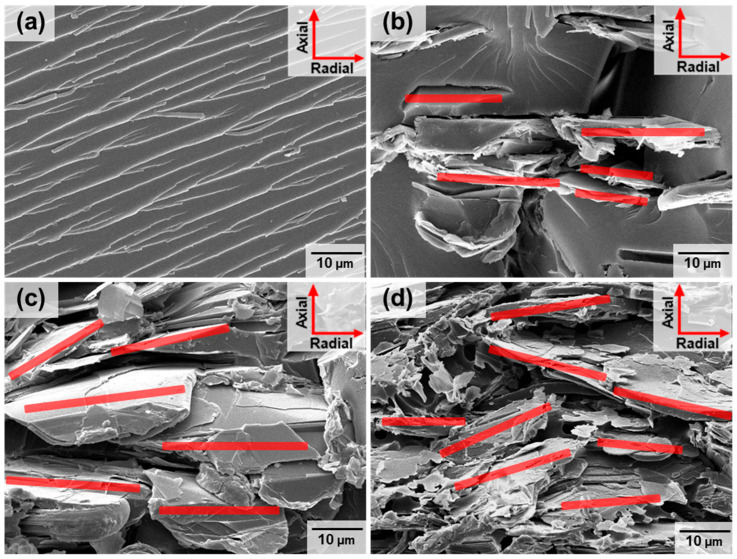
Displayed are representative field emission scanning electron microscope (FE-SEM) images captured from fractured *h*-BN/PHT composites. The red lines represent an aligned *h*-BN in the radial direction. The composites exhibit different *h*-BN volume fractions: (**a**) 0, (**b**) 6, (**c**) 12, and (**d**) 55 vol%. Reproduced from Ref. [61]. Copyright 2023, Elsevier.

The superior thermal conductivity observed is attributed to the exceptional alignment of fillers within the PHT matrix. An optimal filler aspect ratio is achieved when the *h*-BN fillers are perfectly oriented in the radial direction of the sample, as opposed to a random or axial orientation. Radial (K_//_) and axial (K_⊥_) thermal conductivities are measured using the transient plane source method, indicating a highly aligned *h*-BN within the sample in the radial direction (Figure 5c). The degree of filler orientation is estimated using the relationship (K_//_ − K_⊥_)/(2K_//_ + K_⊥_), in which the denominator is the sum of K in all directions and can allow for the assessment of how aligned the material is in the radial direction compared to the axial direction [59,60,61,62]. The estimated filler alignment as a function of the filler loading is provided in Figure 5d. The estimated filler alignment would give 0.5 for the perfect filler orientation in the in-plane direction. As depicted in Figure 5d, all composite materials, even those with the lowest filler loading investigated in this study, display a pronounced orientation of *h*-BN along the radial direction of the sample. To verify the alignment of the filler, scanning electron microscopy is utilized for a direct examination of cross-sections from selected samples with varying *h*-BN loadings. The field emission scanning electron microscope (FE-SEM) images, presented in Figure 6, unequivocally validate the consistent radial alignment of fillers regardless of filler content across all samples. This observational method serves to avoid redundancy and ensures a comprehensive understanding of the filler distribution in composite materials.

It becomes evident that the nanocomposite fabrication of the vitrimer matrix PHT, with the assistance of flattened molecules, allows for facile radial orientation even with a minimal amount of added *h*-BN. Consequently, the establishment of filler networks occurs, creating an elongated heat transfer pathway and minimizing phonon scattering [60]. In summary, it can be conclusively asserted that the vitrimer PHT, in stark contrast to traditional polymers, plays an active and influential role in influencing the orientation of fillers within the composite material. The potential and expectations for achieving heightened filler alignment in composite materials are anticipated through the incorporation of diverse fillers, except *h*-BN, exhibiting future anisotropic characteristics. As such, the exploration of anisotropy in composites by introducing various fillers holds promise for advancing the field, offering avenues for improved thermal conductivity in heat dissipation composites.

## 3. Reprocessability and Recyclability of Vitrimer-Assisted Filler Nanocomposites

**Figure 7:**
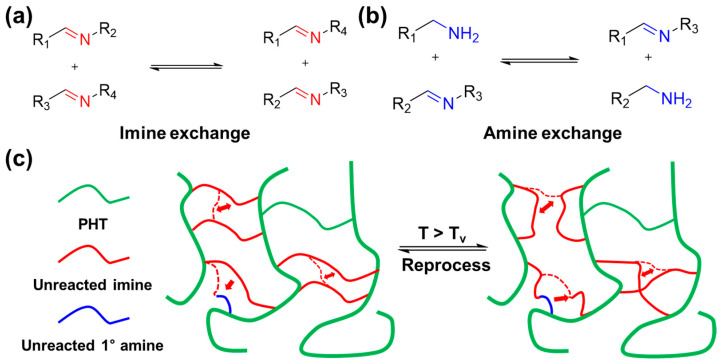
(**a**) Schematic representation of the imine exchange reaction, and (**b**) transamination providing the chemical rearrangements characterizing this reversible reaction. (**c**) Schematic illustration of the crosslink network rearrangement by dynamic bond exchange reactions over T_v_.

From the research literature, findings unveil the presence of unreacted imines and primary amines [63]. This discovery implies the potential of PHT to exhibit vitrimer behavior through two dynamic bond exchange reactions. The first involves imine metathesis, occurring between imines, while the second is transamination, which encompasses the exchange between amines and imines, as illustrated in the respective reactions, as shown in Figure 7. Above the temperature of T_v_, dynamic exchange reactions occur, making reprocessing possible.

**Figure 8:**
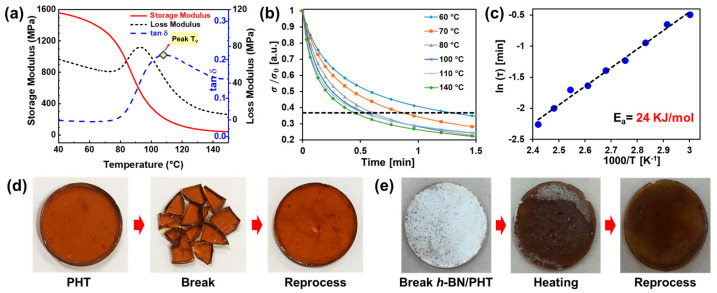
(**a**) Dynamic mechanical analysis (DMA) is employed to investigate the behavior of PHT up to 150 °C. (**b**) The stress relaxation curves for poly(hexahydrotriazine) (PHT) across temperatures up to 140 °C. The dotted line indicates constant (e^−1^). (**c**) Characteristic relaxation times (τ) for neat PHT are determined as part of the analysis. Photographs illustrate the preparation of a disk-shaped sample, its subsequent fragmentation into smaller pieces, and the reprocessing of (**d**) PHT and (**e**) *h*-BN/PHT composites. Reproduced from Refs. [61,63]. Copyright 2023, Elsevier and Wiley.

Identifying the T_v_ temperature is crucial, as it plays a significant role in the reformation of vitrimer composites through exchange reactions. Determining this temperature is accomplished using a dynamic mechanical analysis (DMA), where tan delta reveals the point at which exchange reactions occur [63]. Additionally, the relatively low activation energy (E_a_) of vitrimers was obtained through Arrhenius plots in relaxation tests using DMA (Figure 8b,c) [63,64]. The characteristic relaxation time *τ* follows Arrhenius’ law and fits the Arrhenius equation upon variation in the temperature, as per the following Equation (2):

(2) $$\tau =\frac{1}{K}exp(\frac{E_{a}}{RT})\;$$

where *K* is the reaction constant, *R* is the ideal gas constant, and *T* is the temperature (K). The linear relationship between the characteristic relaxation time and the temperature for each system was obtained by linear fitting of the Arrhenius equation, wherein the slopes of the straight lines give activation energies of 24 kJ/mol, which makes them easily reprocessable. Consequently, after creating the composite, reprocessing leads to reshaping, as illustrated in Figure 8d. Similarly, reshaping occurs even when *h*-BN is mixed, demonstrating the reformation through dynamic exchange reactions (Figure 8e) [63]. They can be easily reshaped at temperatures above the designated T_v_ threshold.

**Figure 9:**
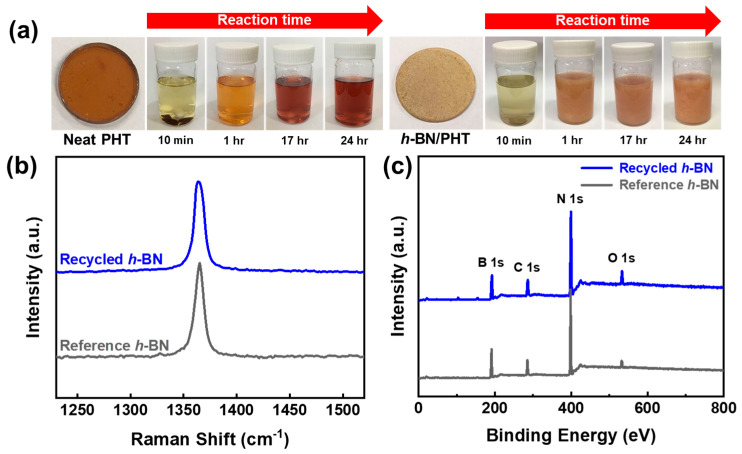
(**a**) PHT and *h*-BN/PHT composites undergo depolymerization by immersing them in an acidic aqueous solution (pH = 2) at room temperature for one day. (**b**) Raman spectra are then compared between the original *h*-BN (blue) and the recovered *h*-BN from the *h*-BN/PHT composite (gray). (**c**) XPS spectra of the recycled *h*-BN are compared with the reference *h*-BN, with normalization based on the maximum N1s peak intensity of each scan. Reproduced from Ref. [61]. Copyright 2023, Elsevier.

The PHT matrix was employed to reclaim *h*-BN from composite materials through the chemical breakdown of PHT in low pH conditions (≤2) [61,63]. After soaking PHT in an acidic solution for more than 24 h, the absence of any solid residue affirmed the complete chemical breakdown (Figure 9a). Breaking down composite materials in an acidic solution resulted in a translucent pink mixture, from which the *h*-BN filler, surpassing 99% in weight, easily separated after multiple washes and vacuum drying. Assessing the recovered *h*-BN’s quality involved comparing values from Raman spectra (14.9 cm^−1^ and 15.0 cm^−1^), indicating a sustained quality (Figure 9b). This was corroborated by XPS results, revealing similarities in the elemental composition (Figure 9c). Furthermore, PHT withstood dissolution in common organic solvents even after prolonged soaking, demonstrating a resilient resistance, with decomposition only occurring under acidic conditions. In conclusion, the nanocomposite for heat dissipation, leveraging the characteristics of the vitrimer PHT, stands as an advanced vitrimer with facile reprocessability and recyclability.

## 4. Natural Supramolecule-Based Vitrimer Nanocomposites Containing a Large Thermal Pathway

Tannic acid (TA), with its bio-based polyphenolic structure, can be considered a polymeric compound. Its polymer-like properties arise from the presence of multiple phenolic hydroxyl groups in its structure, allowing it to form complex networks through various interactions. Tannic acid is especially known for its ability to form strong and stable crosslinks [65,66]. This property is particularly useful in polymer chemistry, where crosslinking enhances the mechanical strength and stability of polymers. The phenolic hydroxyl groups in tannic acid can react with various substrates, creating a crosslinked network [67]. The intricate network of tannic acid can be utilized to form a large thermal pathway, enhancing the production of a nanocomposite with high thermal conductivity. Boronic ester bonds make vitrimers unique and distinguish them from traditional polymers, as they provide the material with properties like reprocessability, stress relaxation, and adaptability to change conditions [24,25,26]. We introduce the incorporation of tannic acid’s phenolic network in boronic ester vitrimers, which can create large thermal pathways.

**Figure 10:**
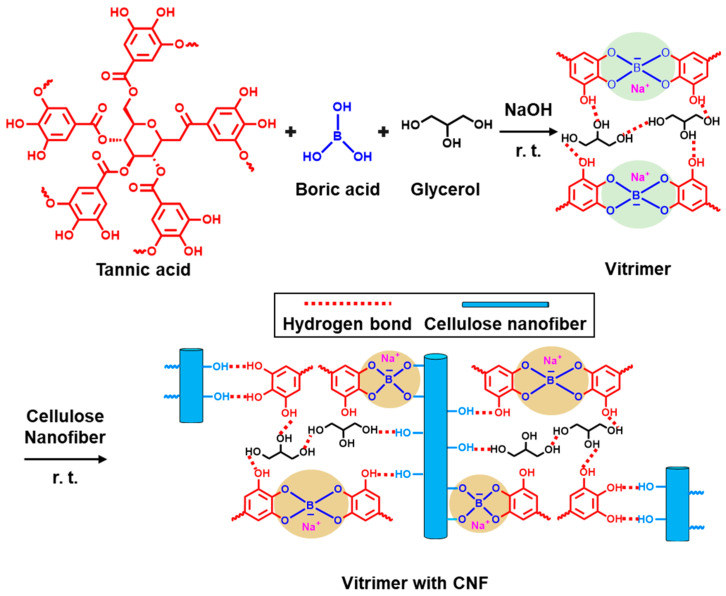
Schematic diagram of the chemical structures engaged in the creation of crosslinked networks in a vitrimer derived from natural sources. Reproduced from Ref. [68]. Copyright 2023, Elsevier.

To create a vitrimer with a high crosslink density, tannic acid, boron acid, and glycerol are utilized under base conditions (OH^−^ generated by a NaOH solution) to form borate ions [68]. Subsequently, a vitrimer incorporating boronic ester bonds is generated. During the optimization process (Table 1), a vitrimer with an increased crosslink density is formed to enhance the thermal pathway and reduce phonon scattering during manufacturing. In addition to enhancing the thermal pathway, the nanocomposite, glycerol, and cellulose nanofibers (CNFs) are mixed to enable intermolecular hydrogen bonding. CNFs, a nano-scale filler commonly used to enhance composite properties [69], are included in the system. A new system is prepared by adding CNF, aiming to create a constant structure of CNFs with abundant hydroxyl groups, facilitating the formation of hydrogen bonds with both boric acid and tannic acid (refer to Figure 10).

**Figure 11:**
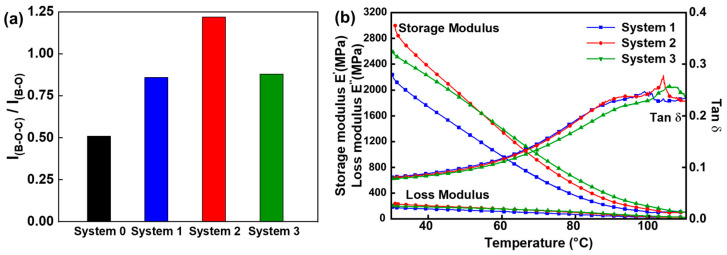
(**a**) The intensity ratio of FT-IR peaks from B-O-C to B-O for composites in systems 0–3. (**b**) Dynamic mechanical analysis plots for composites in systems 1, 2, and 3. Reproduced from Ref. [68]. Copyright 2023, Elsevier.

The results, as confirmed by FT-IR, indicate that system 2, which includes CNFs, forms the highest proportion of dynamically shared bonds, as shown in Figure 11a,b [68]. Moreover, a DMA analysis reveals that system 2 exhibits the highest glass transition temperature (T_g_), and when calculating the crosslink density [68], it shows the highest value (0.0093 mol/cm^3^ for system 2) (Figure 11b). Therefore, leveraging the high crosslink density boronic ester-based vitrimer system 2, it is utilized for the production of a high-thermal conductivity nanocomposite.

**Figure 12:**
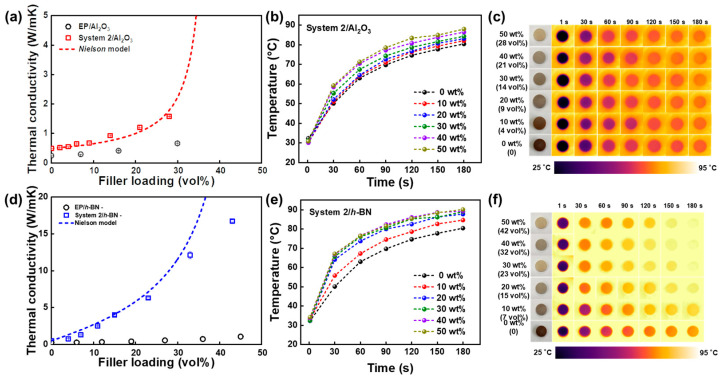
A graph of experimental and theoretical thermal conductivity of (**a**) system 2/Al_2_O_3_. An increase in the surface temperatures of system 2/Al_2_O_3_ composites over time during heating, along with (**b**) corresponding infrared thermal depictions for the composites incorporating different Al_2_O_3_ concentrations. (**c**) Variations in the surface temperatures of system 2/*h*-BN composites during heating. Measured results in the same sequence corresponding to System 2/*h*-BN composites (**d**–**f**). Reproduced from Ref. [68]. Copyright 2023, Elsevier.

In the pursuit of potential applications in thermal management materials, composite materials were created by blending the system 2 vitrimer with highly thermally conductive fillers such as Al_2_O_3_ and *h*-BN to advance its thermal conductivity [68]. The findings related to thermal conductivity, as depicted in Figure 12, showcase the thermal conductive properties of the composites created using system 2 with varying proportions of the Al_2_O_3_ and *h*-BN fillers. Significantly, the thermal conductive characteristics of the composite escalated in direct correlation with the filler content. Without fillers, the unaltered System 2 composite exhibited a thermal conductivity of 0.49 Wm^−1^K^−1^, representing a twofold increase compared to the thermal conductivity of the pure bisphenol A epoxy resin (0.24 Wm^−1^K^−1^). The thermal conductivity of system 2/Al_2_O_3_ rose to 1.58 Wm^−1^K^−1^ with the inclusion of 28 vol% of Al_2_O_3_, marking a threefold increase compared to the only system 2 composite and a twofold increase compared to the epoxy composite containing 28 vol% of Al_2_O_3_ (0.65 Wm^−1^K^−1^). Similarly, the system 2/*h*-BN composite achieved a remarkable thermal conductivity of 16.75 Wm^−1^K^−1^, containing 43 vol% of *h*-BN. This measurement represents a 34-time increase compared to the thermal conductivity of only the system 2 composite and a 16-time increase compared to the epoxy composite containing 43 vol% of *h*-BN (1.04 Wm^−1^K^−1^) [68]. A comparison between the theoretical (*Nielsen* model) and experimental data for the system 2/Al_2_O_3_ and *h*-BN composites is depicted in Figure 12a,d. Both sets of data show a progressive elevation in filler concentrations, indicating that optimizing the filler loading can improve thermal conductivity [59,61,68]. The thermal conductive properties of system 2 and its composite surpass those of commercially accessible epoxy mold compounding materials, as evident from these outcomes.

Furthermore, cylindrical composites (20 mm in diameter, 4 mm in height) composed of system 2/Al_2_O_3_ and system 2/*h*-BN (with a 0–50 weight% filler) were positioned on a heating plate at a consistent temperature of 90 °C. The heat conductive characteristics of every composite were directly examined employing an infrared thermal imaging tool, where brighter colors signaled elevated temperatures on the surface of the composite. Figure 12b,c,e,f show temperature changes in the system 2/Al_2_O_3_ and *h*-BN composites from 1 to 180 s, throughout an identical duration. A persistent trend is observed, where augmented filler concentrations consistently result in elevated surface temperatures in all composite systems, underscoring the contribution of fillers in enhancing the heat transfer and thermal conductive properties [68].

**Figure 13:**
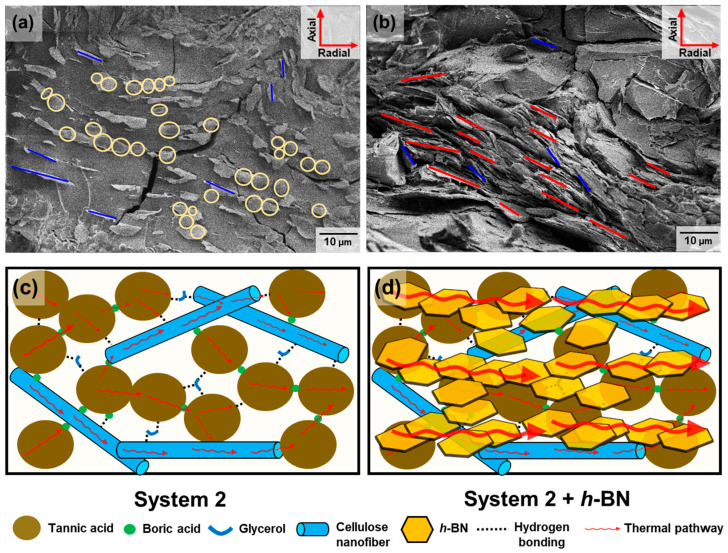
Cross-sectional FE-SEM images of (**a**) system 2 Al_2_O_3_ and (**b**) *h*-BN. The yellow circles represent Al_2_O_3_, the blue lines represent CNF, and the red lines represent *h*-BN. Mechanism of thermal passway established by the crosslinking networks involving hydrogen bonds and boronic ester bonds in composites of (**c**) system 2 and (**d**) the composites subsequent to the incorporation of the *h*-BN filler into system 2. Reproduced from Ref. [68]. Copyright 2023, Elsevier.

To understand the roles of TA and CNF in achieving this high thermal conductivity, the researcher examined the cross-sectional morphologies of system 2 + 30 wt% of Al_2_O_3_ and system 2 + 30 wt% of *h*-BN using FE-SEM, as shown in Figure 13a and b, respectively. The results reveal that the CNF, with a high aspect ratio, was uniformly dispersed in system 2, leading to increased crosslinking and a greater number of hydrogen bonds. This enhanced the thermal pathways for phonon vibration, reducing phonon scattering.

Furthermore, this confirmed that the fillers were horizontally oriented due to the vitrimer nature of system 2. This unique alignment, influenced by the vitrimer’s elastic properties, resulted in a longer heat transfer pathway, reduced phonon scattering, and higher thermal conductivity. In particular, the plate-shaped *h*-BN exhibited a clearer alignment in the horizontal direction, with filler–filler interconnections contributing to an even larger percolation network. The high dispersibility of the filler and the formation of a good percolation network were identified as crucial factors contributing to the high thermal conductivities observed in these filler-containing composites.

These findings propose a mechanism whereby the high thermal conductivity in these composites is due to the structure of system 2. TA and CNF, characterized by their large size and aromatic/cyclic structures, with plate and rod-like shapes, have excellent thermal conductivities. The many hydroxyl groups on their surfaces create an effective network through B-O-C or hydrogen bonding, establishing the required thermal pathway for high thermal conductivity. Furthermore, the vitrimer with *h*-BN forms a highly horizontally oriented plate-like layer during the thermal process, further enhancing thermal conductivity (Figure 13c).

## 5. Thermal Grating Structure Using Reprocessability of Vitrimer

**Figure 14:**
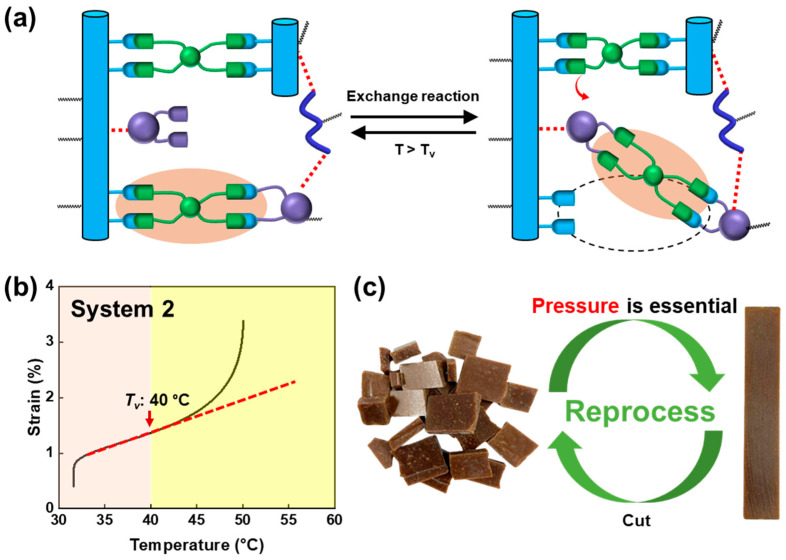
(**a**) Schematic representation of the network rearrangement process through a boronic exchange reaction. (**b**) The topological freezing transition temperatures (T_v_) for system 2, as determined through the creep test. (**c**) Reprocessing capability of system 2 utilizing a heating press. Reproduced from Ref. [68]. Copyright 2023, Elsevier.

The boronic ester functional group exhibits dynamic exchange reactions, particularly through its boronic ester bonds. Determining the reprocessing temperature (T_v_) is crucial for these bonds. Various techniques exist to evaluate T_v_, and an innovative method involves employing the creep test [68]. During this test, the slope of strain values increases non-linearly at a specific temperature, indicating a balance between the vitrimer intermolecular bond breakage and recombination rates as the temperature rises (see Figure 14a). The temperature at which the slope changes non-linearly signifies T_v_. In Figure 14b, T_v_, for the current vitrimer system, is approximately 40 °C, and this value remains constant irrespective of the content of the CNF. Notably, this thermally conductive composite cannot be reprocessed and reshaped without external pressure. However, it becomes feasible under pressure above the T_v_ temperature. Subsequently, the reprocessed composite sample is fabricated using the heat-press method, as depicted in Figure 14c.

**Figure 15:**
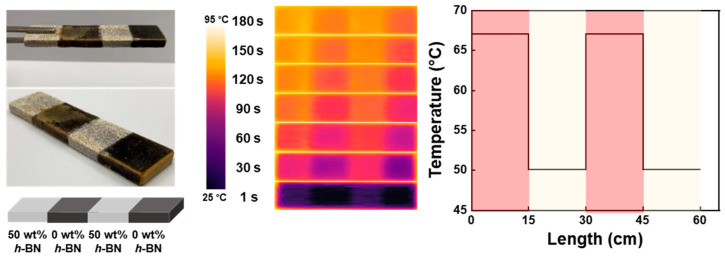
Photographic images of the reprocessing thermal grating composite, thermal infrared images, and a temperature-change graph along the length depict the composite consisting of four segments: system 2/*h*-BN (50 wt%) and system 2 in succession. Reproduced from Ref. [68]. Copyright 2023, Elsevier.

In Figure 15, the advantage of reformation is solely utilized through the thermal pressure molding of a composite grating, formed by connecting four segments—system 2 and system 2/*h*-BN (50 wt%) repeatedly—using the reversible properties of the boronic ester bonds under heat-press conditions. Placed on a heating plate at 90 °C, it showcases the surface temperature change along the sample’s longitudinal direction, affirming the successful creation of a four-segment composite grating with varying thermal conductivities. In conclusion, utilizing the advantages of heat-pressure reprocessing allows for the easy creation of a grating structure. This enables efficient heat transfer only in the desired areas, facilitating effective reprocessing.

## 6. Recyclability of Vitrimer and Sustainability of Vitrimer Nanocomposites

**Figure 16:**
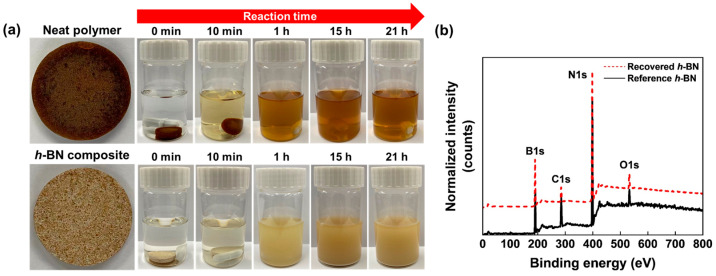
(**a**) The specimens were dissolved by immersion. Top row, the pristine matrix; bottom row, the *h*-BN composite. (**b**) XPS spectra of the recovered and reference *h*-BN filler samples. The peaks were normalized using the maximum N1s peak intensity of each scan. Reproduced from Ref. [68]. Copyright 2023, Elsevier.

The composite showcased notable dissolution in a citric acid solution after 21 h, stemming from the hydrolysis of boronic ester bonds that led to the collapse of the crosslinked network [68,70]. While achieving complete solubility in water after 96 h, the composite remained non-soluble in ethanol. These observations underscore the distinct solubility characteristics of the prepared composites in contrast to conventional thermosetting materials. As depicted in Figure 16a, pristine vitrimer compounds demonstrated the ability to dissolve in acidic solutions. Utilizing these features, the recyclability of a thermally conductive composite filled with *h*-BN was assessed by immersing it in a 1 mol/L citric acid solution. Within 21 h, the composite underwent total dissolution in the citric acid solution, as illustrated in Figure 16a. The non-soluble white powder, indicative of the *h*-BN filler, was effortlessly isolated via filtration, proceeded by iterative rinsing with deionized water and acetone and subsequent vacuum drying. The recovered *h*-BN and the reference *h*-BN underwent XPS analysis (Figure 16b). The results indicate that both samples exhibited comparable elemental compositions, confirming the successful recycling of the filler without substantial alterations to its composition.

## 7. Conclusions

Vitrimers, as covalent adaptable network polymers, have opened new avenues in materials science, offering a unique blend of stability and processability. The ability of vitrimers to undergo dynamic covalent bond exchange reactions, responding to external stimuli, has paved the way for applications in various industries. In particular, their role in high thermal-conductivity composite materials is significant, contributing to developing alternative polymers instead of traditional composites. From the viewpoint of thermal conductivity, the incorporation of vitrimers into composite fillers can help in directional alignment, forming well-structured intermolecular networks that effectively minimize phonon scattering. The potential and expectations for the further enhancement of filler alignment when creating composites by incorporating different fillers with future anisotropic characteristics are also anticipated. In addition, by utilizing natural materials, vitrimers create a broad heat passway for phonons through dynamic network interactions, resulting in significantly enhanced thermal conductivity compared to conventional thermosetting polymers.

Moreover, the dynamic exchange reactions of vitrimers enable the formation of easy reprocessing that can adapt to external forces. This distinctive feature allows damaged or fractured composite materials to undergo reuse through reprocessing. The ability to break crosslinks presents the potential for recycling both the matrix and filler components of vitrimer high thermal conductive composites. The material’s adaptability to external forces, combined with the capability for reshaping and reprocessing, underscores its potential to develop high-performance and recyclable composite materials, surpassing the thermal conductivity of traditional thermosetting polymers.

In the evolving field of industrial devices, as they trend towards becoming smaller, thinner, and lighter, effective heat dissipation is anticipated to emerge as a critical challenge. Addressing these concerns, the advantages of vitrimers, as highlighted in this review, position them as a promising solution for designing polymer composite materials with a focus on efficient heat dissipation and sustainable alternatives.

## Figures and Tables

**Table 1:** Optimization of the natural supramolecule-based vitrimer system 0, 1, 2, and 3 composites from Ref. [68].

	System 0	System 1	System 2	System 3
Tannic acid (g)	3	3	3	3
Boric acid (g)	0.6	0.6	0.6	0.6
Glycerol (mL)	0.1	0.1	0.1	0.1
NaOH (mL)	−	3.5	3.5	3.5
Cellulose nano-fiber	−	−	0.6	0.6

## Data Availability

Data are contained within the article.
